# Quantitative genomics of locomotor behavior in *Drosophila melanogaster*

**DOI:** 10.1186/gb-2007-8-8-r172

**Published:** 2007-08-21

**Authors:** Katherine W Jordan, Mary Anna Carbone, Akihiko Yamamoto, Theodore J Morgan, Trudy FC Mackay

**Affiliations:** 1 Department of Genetics and WM Keck Center for Behavioral Biology, North Carolina State University, Raleigh, NC 27695-7614, USA; 2 Division of Biology, Kansas State University, Ackert Hall, Manhattan, KS 66506, USA

## Abstract

**Background:**

Locomotion is an integral component of most animal behaviors, and many human health problems are associated with locomotor deficits. Locomotor behavior is a complex trait, with population variation attributable to many interacting loci with small effects that are sensitive to environmental conditions. However, the genetic basis of this complex behavior is largely uncharacterized.

**Results:**

We quantified locomotor behavior of *Drosophila melanogaster *in a large population of inbred lines derived from a single natural population, and derived replicated selection lines with different levels of locomotion. Estimates of broad-sense and narrow-sense heritabilities were 0.52 and 0.16, respectively, indicating substantial non-additive genetic variance for locomotor behavior. We used whole genome expression analysis to identify 1,790 probe sets with different expression levels between the selection lines when pooled across replicates, at a false discovery rate of 0.001. The transcriptional responses to selection for locomotor, aggressive and mating behavior from the same base population were highly overlapping, but the magnitude of the expression differences between selection lines for increased and decreased levels of behavior was uncorrelated. We assessed the locomotor behavior of ten mutations in candidate genes with altered transcript abundance between selection lines, and identified seven novel genes affecting this trait.

**Conclusion:**

Expression profiling of genetically divergent lines is an effective strategy for identifying genes affecting complex behaviors, and reveals that a large number of pleiotropic genes exhibit correlated transcriptional responses to multiple behaviors.

## Abstract 2

The locomotor behavior of Drosophila melanogaster was quantified in a large population of inbred lines derived from a single natural population, showing that many pleiotropic genes show correlated transcriptional responses to multiple behaviors.

## Background

Locomotion is required for localization of food and mates, escape from predators, defense of territory, and response to stress, and is, therefore, an integral component of most animal behaviors. In humans, Parkinson's disease, Huntington's disease, activity disorders and depression are associated with deficits in locomotion. Thus, understanding the genetic architecture of locomotor behavior is important from the dual perspectives of evolutionary biology and human health.

Locomotion is a complex behavior, with variation in nature attributable to multiple interacting quantitative trait loci (QTL) with individually small effects, whose expression is sensitive to the environment [1]. Dissecting the genetic architecture of complex behavior is greatly facilitated in model organisms, such as *Drosophila melanogaster*, where one can assess the effects of mutations to infer what genes are required for the manifestation of the behavior, and map QTL affecting naturally occurring variation with high resolution [2]. General features of the genetic architecture of complex behaviors are likely to be recapitulated across diverse taxa. Basic biological processes, including the development of the nervous system, are evolutionarily conserved between flies and mammals [3]. Thus, orthologues of genes affecting *Drosophila *locomotion may well be relevant in humans. For example, Parkinson's disease is associated with progressive degeneration of nigrostriatal dopaminergic neurons [4,5], and dopamine has also been implicated in locomotion of mice [6] and *Drosophila *[1,7-12].

Several studies reveal the underlying genetic complexity of locomotor behavior in *Drosophila*. The neurotransmitters serotonin (5-hydroxytryptamine) [13], octopamine (the invertebrate homolog of noradrenaline) [14], and γ-aminobutyric acid [15] affect *Drosophila *locomotion; as do genes required for the proper neuroanatomical development of the mushroom bodies and components of the central complex, brain regions required for normal locomotion [16-21]. Recently, we developed a high-throughput assay to quantify the 'locomotor reactivity' component of locomotor behavior (measured by the level of activity immediately following a mechanical disturbance), and used this to map QTL segregating between two inbred lines that had significantly different levels of locomotor reactivity [1]. We identified 13 positional candidate genes corresponding to the QTL. Three of these genes were known to affect adult locomotion; six had mutant phenotypes consistent with an involvement in regulating locomotion, although effects on locomotor behavior were not quantified previously; and the remaining four genes, all encoding RNA polymerase II transcription factors implicated in nervous system development, were novel candidate genes affecting locomotor behavior. This study highlights the power of using natural allelic variants to study complex behavior [22], but was limited to identifying genes segregating in the two parental lines used, which represent a restricted sample of alleles segregating in a natural population.

An alternative strategy to discover genes affecting complex behaviors is to combine artificial selection for divergent phenotypes with whole genome expression profiling [23-28]. The rationale of this approach is that genes exhibiting consistent changes in expression as a correlated response to selection are candidate genes affecting the selected trait. This strategy has two advantages compared to traditional QTL mapping paradigms and unbiased screens for mutations affecting behavioral traits. First, initiating artificial selection from a large base population recently derived from nature ensures that a larger and more representative sample of alleles affecting segregating variation in behavior is included than in QTL mapping studies utilizing two parental lines. Second, assessing the behavioral effects of mutations in candidate genes whose expression is co-regulated in the genetically divergent lines is more efficient than unbiased mutational screens for identifying genes affecting the trait of interest [23,26,27]. Here, we have combined this strategy with classical quantitative genetic analysis to further understand the genetic architecture of locomotor reactivity. We created artificial selection lines from a genetically heterogeneous background and selected for 25 generations to derive replicate lines with increased and decreased levels of locomotor reactivity, as well as unselected control lines. We also measured locomotor reactivity in a population of 340 inbred lines derived from the same natural population. We then used whole genome expression profiling to quantify the suite of genes that were differentially expressed between the selection lines. Functional tests of mutations in ten of the differentially expressed genes identified seven novel candidate genes affecting locomotor behavior.

## Results

### Natural genetic variation in locomotor reactivity

**Figure 1:**
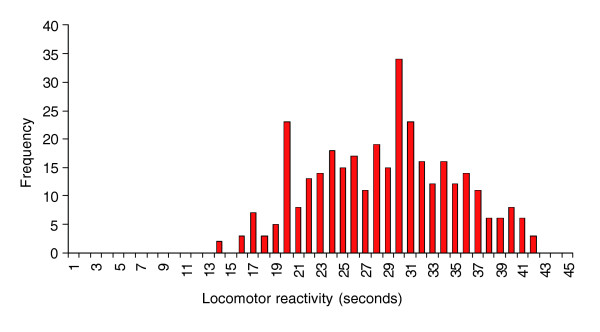
Frequency distribution of locomotor reactivity scores (in seconds) among inbred lines derived from the Raleigh population.

We quantified the magnitude of variation in locomotor activity among a panel of 340 inbred lines derived from the Raleigh natural population. We observed substantial naturally segregating variation in locomotor reactivity behavior (F_326,25736 _= 41.84, *P *< 0.0001; Figure 1). The estimate of broad-sense heritability (*H*^2^) of locomotor reactivity in this population was high: *H*^2 ^= 0.519. The line by sex interaction term was not significant (F_339,25736 _= 0.11, *P *= 1.0000), indicating that magnitude and/or rank order of the sexual dimorphism does not vary among the lines in this population. The correlation in locomotor reactivity between the sexes (*r*_*GS *_= 0.973 ± 0.015) was correspondingly high and positive, and not significantly different from unity.

### Response to artificial selection for locomotor reactivity

**Figure 2:**
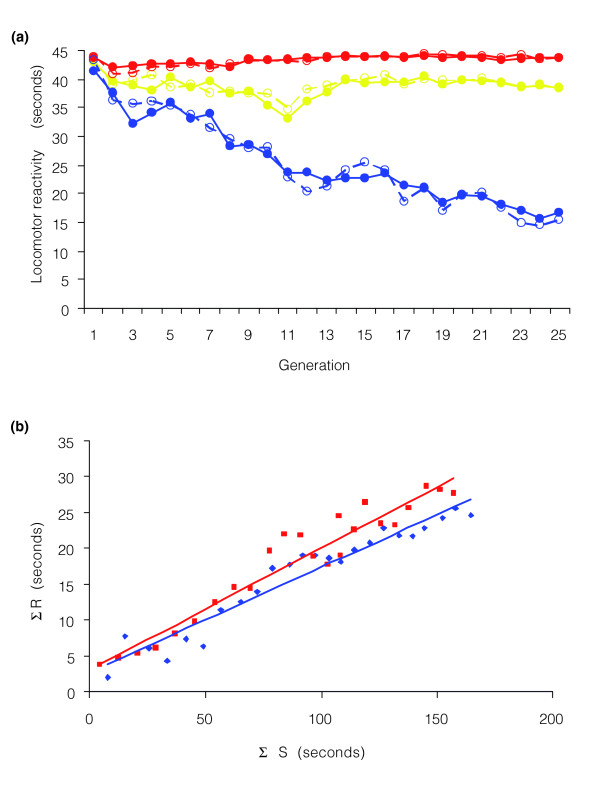
Phenotypic response to selection for locomotor reactivity. **(a) **Mean activity scores of selection lines (in seconds). The blue dots represent the L lines, the yellow dots represent the C lines, and the red dots represent the H lines. Solid lines and filled circles, replicate 1; dashed lines and open circles, replicate 2. **(b) **Regressions of cumulative response on cumulative selection differential for divergence between H and L lines. The blue diamonds and blue line represent replicate 1, and the red squares and red line represent replicate 2.

We derived a heterogeneous base population from isofemale lines sampled from the Raleigh natural population, and used artificial selection to create replicate genetically divergent lines with high (H) and low (L) activity levels, as well as replicate unselected (control, C) lines. At generation 25, the H and L lines diverged by 27.6 seconds, or 61% of the total 45 s assay period (Figure 2a).

We estimated realized heritability (*h*^2 ^± standard error of the regression coefficient) of locomotor reactivity from the regressions of the cumulated response on cumulated selection differential [29]. Heritability estimates from the divergence between H and L lines over 25 generations were *h*^2 ^= 0.147 ± 0.008 (*P *< 0.0001) for replicate 1 and *h*^2 ^= 0.170 ± 0.010 (*P *< 0.0001) for replicate 2 (Figure 2b). The selection response was asymmetrical, largely due to low selection differentials in the H lines. Estimates of realized heritability for each of the selection lines (estimated as deviations from the contemporaneous control) were *h*^2 ^= 0.030 ± 0.036 (*P *= 0.43) and *h*^2 ^= 0.074 ± 0.0265 (*P *= 0.01) for H line replicates 1 and 2, respectively; and *h*^2 ^= 0.181 ± 0.0093 (*P *< 0.0001) and *h*^2 ^= 0.201 ± 0.011 (*P *< 0.0001) for L line replicates 1 and 2, respectively. There was no inbreeding depression for locomotor reactivity: the regression of locomotor behavior in the control lines over 25 generations was *b *= 0.0006 ± 0.053 (*P *= 0.98) and *b *= -0.012 ± 0.044 (*P *= 0.78) for C line replicates 1 and 2, respectively.

### Correlated phenotypic response to selection for locomotor reactivity

**Figure 3:**
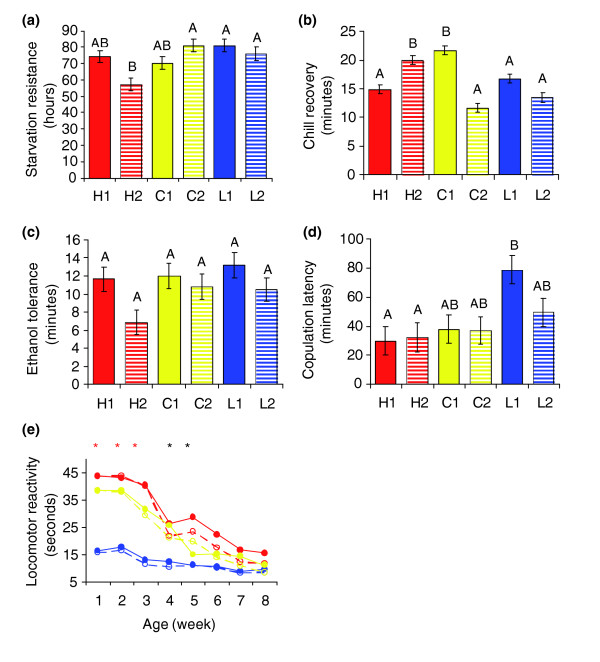
Correlated phenotypic responses to selection. All scores are pooled across three successive generations. Lines with the same letter are not significantly different from one another at *P *< 0.05. H lines are red, C lines are yellow, L lines are blue. Solid lines and bars represent replicate 1, and dashed bars and lines denote replicate 2. The red asterisk denotes each line is significantly (*P *< 0.05) different from each other, and the black asterisk denotes H lines and C lines are not significantly different from each other, but are significantly different than L lines. **(a) **Starvation resistance, **(b) **chill coma recovery, **(c) **ethanol tolerance, **(d) **copulation latency, **(e) **behavioral locomotor senescence.

We evaluated whether the response to selection was specific for locomotor activity in response to a mechanical stress, or if other traits involved in stress response or behaviors that have a locomotor component were also affected. We did not observe significant differences among the selection lines for starvation resistance (F_2,3 _= 1.22, *P *= 0.41; Figure 3a), chill coma recovery (F_2,3 _= 0.13, *P *= 0.89; Figure 3b), ethanol sensitivity (F_2,3 _= 0.73, *P *= 0.55; Figure 3c), or copulation latency (F_2,3 _= 4.21, *P *= 0.13; Figure 3d). These results suggest that the response to selection is specific for locomotor reactivity, and not a general behavioral response; that is, the slowly reacting low activity lines are not generally 'sick'.

We assessed whether selection for increased and decreased locomotor reactivity early in life affected locomotion at later ages - that is, whether selection affected the typical senescent decline in locomotor behavior with age [30]. We repeated our assay of locomotor reactivity on the selection lines each week until the flies were eight weeks old. We found that by week 4 (F_2,3 _= 8.76, *P *= 0.05; Figure 3e) the H and C lines no longer differed, and by week 6 (F_2,3 _= 3.33, *P *= 0.18; Figure 3e), none of the lines differed from one another. Thus, the selection response was specific for genes affecting locomotor reactivity of young animals. We infer from this result that either there is little genetic variation for locomotor reactivity in aged flies, or that such variation is genetically uncoupled from that which affects locomotion of young flies.

### Transcriptional response to selection for locomotor reactivity

We assessed transcript abundance in the H, L, and C selection lines using Affymetrix high density oligonucleotide whole genome microarrays, for flies of the same age and physiological state as selected individuals. The raw microarray data are given in Additional data file 1, and have been deposited in the GEO database [31] under series record GSE5956 [32]. We used factorial ANOVA to quantify statistically significant differences in transcript level for each probe set on the array. Using a false discovery rate [33] of *Q *< 0.001, we found 8,766 probe sets were significant for the main effect of sex, 1,825 were significant for the main effect of line, and 42 were significant for the line × sex interaction (Additional data file 2). All 42 probe sets that were significant for the interaction term were also significant for the main effect of line.

**Figure 4:**
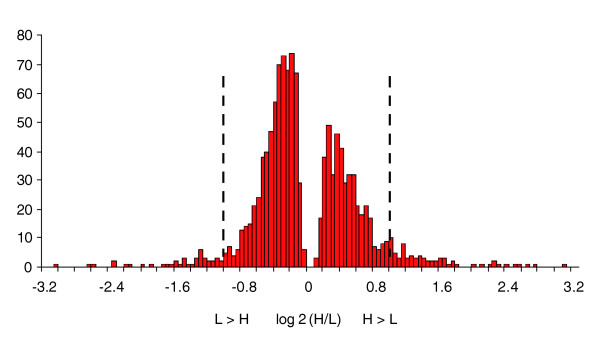
Frequency of relative fold-change of probe sets with significant changes in transcript abundance between H and L selection lines, pooled over sexes. The vertical dashed black lines demarcate two-fold changes in transcript abundance.

We used ANOVA contrast statements on the 1,825 probe sets with differences in transcript abundance between selection lines to detect probe sets that were consistently up- or down-regulated in replicate lines [25,27]. We found 1,790 probe sets (9.5%) that differed between the selection lines when pooled across replicates (Additional data file 3). The pattern of the transcriptional response to selection was complex, and fell into four categories: H ≥ C ≥ L (H > L, 486 probe sets); H ≤ C ≤ L (H < L, 686 probe sets); H ≤ C ≥ L (379 probe sets); and H ≥ C ≤ L (239 probe sets). The first two categories can readily be interpreted as linear relationships between transcript abundance and complex trait phenotype, while for the latter two categories the relationship is quadratic, with the most extreme expression values in the C lines. There are two possible explanations for the apparently non-linear patterns of transcriptional response to selection. First, probe sets in the third category could represent cases in which H and L alleles respond to selection, but harbor polymorphisms in the probes used to interrogate expression levels, thus yielding reduced levels of expression relative to the control. Second, the non-linear patterns could be attributable to changes in expression as a consequence of reduced fitness of the selection lines relative to the control. Although there was a widespread transcriptional response to selection for locomotor reactivity, the magnitude of the changes of transcript abundance was not great, with the vast majority much less than two-fold (Additional data file 3, Figure 4).

The probe sets with altered transcript abundance between the selection lines fell into all major biological process and molecular function Gene Ontology (GO) categories (Additional data file 4). We assessed which categories were represented more frequently than expected by chance, based on representation on the microarray, since the over-represented GO categories are likely to contain probe sets for which transcript abundance has responded to artificial selection. Highlights of the transcriptional response to artificial selection for locomotor reactivity are given in Table 1; the complete list of significantly over-represented categories is given in Additional data file 5. The greatest enrichment in the biological process categories were for genes affecting lipid, cellular lipid, steroid and general metabolism, responses to biotic, abiotic, and chemical stimuli, and defense response and responses to toxins and stress. The molecular function categories of catalytic, monooxygenase and oxidoreductase activity were highly enriched, as were the cellular component categories of vesicular, cell and membrane fractions and microsome. These classifications reflect the striking over-representation of genes in the *cytochrome P-450 *and *Glutathione S tranferase *gene families, genes affecting lipid metabolism, and genes encoding immune/defense molecules.

**Table 1:** Differentially represented Gene Ontology categories

Category	Term	Count*	Percent^†^	*P *value^‡^
**Biological process**	Lipid metabolism	110	6.10	3.10E-09
	Steroid metabolism	41	2.30	9.90E-09
	Cellular lipid metabolism	78	4.30	9.30E-08
	Response to toxin	34	1.90	4.20E-06
	Response to biotic stimulus	94	5.20	7.90E-06
	Transport	309	17.10	9.20E-06
	Defense response	92	5.10	9.60E-06
	Response to chemical stimulus	66	3.60	9.80E-06
	Response to abiotic stimulus	84	4.60	1.20E-05
	Localization	352	19.40	1.40E-05
	Metabolism	799	44.10	1.70E-05
	DNA-dependent DNA replication	22	1.20	3.60E-05
	Establishment of localization	340	18.80	3.70E-05
	Physiological process	1,041	57.50	4.30E-05
	DNA replication	36	2.00	1.60E-04
	Cellular physiological process	958	52.90	2.50E-04
	Secretion	48	2.70	2.80E-04
	Electron transport	74	4.10	3.00E-04
	Response to stress	66	3.60	3.20E-04
	Response to stimulus	191	10.50	3.50E-04
	Secretory pathway	45	2.50	4.30E-04
	Response to endogenous stimulus	31	1.70	7.80E-04
	Response to DNA damage stimulus	28	1.50	8.10E-04
	Sleep	7	0.40	1.00E-03
	Primary metabolism	705	38.90	1.50E-03
	Protein complex assembly	28	1.50	2.60E-03
	Intracellular transport	99	5.50	2.90E-03
	DNA repair	25	1.40	2.90E-03
	Intracellular protein transport	80	4.40	3.90E-03
	Regulation of neurotransmitter levels	26	1.40	4.20E-03
	Cell organization and biogenesis	221	12.20	4.40E-03
	Cellular localization	101	5.60	4.50E-03
	Protein localization	91	5.00	4.60E-03
	Heterophilic cell adhesion	6	0.30	4.70E-03
	Proteolysis	129	7.10	5.20E-03
	Establishment of cellular localization	100	5.50	5.70E-03
	Oxygen and reactive oxygen species metabolism	18	1.00	6.20E-03
	Sulfur metabolism	15	0.80	7.00E-03
	Generation of precursor metabolites and energy	92	5.10	7.50E-03
	Cellular metabolism	711	39.30	7.70E-03
	Neurotransmitter secretion	23	1.30	8.00E-03
	Regulated secretory pathway	23	1.30	8.00E-03
	mRNA export from nucleus	7	0.40	8.40E-03
	Establishment of protein localization	82	4.50	8.90E-03
	Sterol metabolism	10	0.60	9.00E-03
	Macromolecule metabolism	498	27.50	9.70E-03
	Chromosome condensation	9	0.50	1.00E-02
	Nuclear transport	16	0.90	1.00E-02
**Molecular function**	Catalytic activity	639	35.30	3.60E-10
	Monooxygenase activity	38	2.10	1.50E-07
	Oxidoreductase activity	131	7.20	1.40E-06
	Protein binding	693	38.30	2.60E-06
	Transporter activity	208	11.50	1.40E-05
	Electron transporter activity	49	2.70	9.40E-05
	Hydrolase activity	294	16.20	2.70E-04
	Sequence-specific DNA binding	12	0.70	4.40E-04
	Tetrapyrrole binding	16	0.90	1.30E-03
	Heme binding	16	0.90	1.30E-03
	Binding	990	54.70	1.80E-03
	Carbon-carbon lyase activity	14	0.80	2.60E-03
	Electrochemical potential-driven transporter activity	43	2.40	2.80E-03
	Porter activity	43	2.40	2.80E-03
	Calmodulin binding	18	1.00	3.60E-03
	Carbohydrate transporter activity	19	1.00	5.10E-03
	Phosphoric monoester hydrolase activity	36	2.00	6.50E-03
	DNA-directed DNA polymerase activity	10	0.60	6.60E-03
	Sugar porter activity	12	0.70	7.40E-03
	Glutathione transferase activity	11	0.60	8.70E-03
	Sugar transporter activity	13	0.70	1.00E-02
**Cellular component**	Microsome	31	1.70	5.80E-10
	Vesicular fraction	31	1.70	5.80E-10
	Cell fraction	34	1.90	1.20E-09
	Membrane fraction	33	1.80	2.10E-09
	Clathrin coat	9	0.50	3.80E-04
	Replication fork	9	0.50	3.80E-04
	Coated membrane	11	0.60	6.70E-04
	Membrane coat	11	0.60	6.70E-04
	Clathrin vesicle coat	8	0.40	1.10E-03
	Clathrin coated vesicle membrane	8	0.40	1.10E-03
	Golgi apparatus	24	1.30	1.40E-03
	Coated pit	5	0.30	1.50E-03
	Cell	673	37.20	1.60E-03
	Cytoplasmic vesicle membrane	10	0.60	1.70E-03
	Vesicle coat	10	0.60	1.70E-03
	Coated vesicle membrane	10	0.60	1.70E-03
	Cytoplasm	239	13.20	2.90E-03
	Membrane	295	16.30	3.00E-03
	Plasma membrane	83	4.60	3.80E-03
	Alpha DNA polymerase	4	0.20	4.30E-03
	Chromosome	40	2.20	5.00E-03

*Number of genes in the annotation category. ^†^Number of genes in the annotation category/total number of significant genes. ^‡^*P *value from a modified Fisher exact test for enrichment of genes in an annotation category. The cross-classified factors in the 2 × 2 contingency tables are genes in the annotation category versus not in the annotation category, and significant genes versus all genes on the array.

### Functional tests of candidate genes

**Figure 5:**
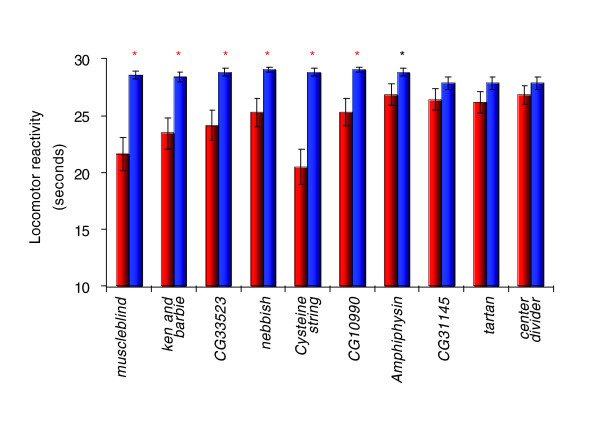
Mean locomotor reactivity scores (seconds) of lines containing *P*-element insertional mutations in candidate genes. The blue bar denotes the Canton S B co-isogenic control line; the red bars indicate the mutant lines. The red asterisk represents mutants that are significantly different from the control line with *P *values that exceed Bonferroni correction for multiple testing (*P *= 0.005), and the black asterisk represents mutants for which *P *< 0.05, but do not surpass the conservative Bonferroni correction.

To assess the extent to which transcript profiling of divergent selection lines accurately predicts genes that directly affect the selected trait, we evaluated the locomotor reactivity of lines containing *P*-element insertional mutations in ten candidate genes that were implicated by the analysis of differential transcript abundance. All of the *P*-element insertions were derived in a common isogenic background, and are viable and fertile as homozygotes [34,35]. The *P*-elements are inserted either in the coding region or approximately 100 bp upstream of the start of transcription of each candidate gene. The candidate genes are involved in diverse biological processes, including signal transduction (*tartan*, *center divider*), neurotransmitter secretion (*Amphiphysin*, *Cysteine string protein*), nervous system and muscle development (*muscleblind*), chromosome segregation (*nebbish*), and copulation (*ken and barbie*). Three of the mutations are in computationally predicted genes (*CG33523*, *CG31145*, and *CG10990*). Six of the mutations exhibited significant differences in locomotor reactivity from the co-isogenic control line, after Bonferroni correction for multiple tests (Table 2, Figure 5). In addition, *Amphiphysin *was formally significant (F_1,112 _= 5.66, *P *= 0.019), but not at the conservative Bonferroni threshold of *P *= 0.005. There was no clear relationship between the pattern of transcriptional response to selection of the candidate genes and the results of the functional tests. The significant genes belonged to categories 1 (H > L, *CG33523 *and *Amphiphysin*), 2 (H < L, *ken and barbie *and *nebbish*) and 3 (H ≤ C ≥ L, *muscleblind*, *Cysteine string protein *and *CG10990*) (Additional data file 3). All of the non-significant candidate genes belonged to category 1. From these data, we infer that transcripts in category 3 do not solely represent instances of changes in expression as a consequence of reduced fitness of the selection lines relative to the control, as in this case one would not expect the genes to affect the selected trait.

**Table 2:** Functional tests of candidate genes

Line	Gene	Mean locomotor reactivity (± SE)	F_1,112_	*P *value	Human ortholog
BG01127	*muscleblind*	21.63 ± 1.47	37.47	< 0.0001	*MBLN1*
BG01259	*Ken and barbie*	23.43 ± 1.32	17.26	< 0.0001	N/A
BG01697	*CG33523*	24.13 ± 1.30	22.08	< 0.0001	N/A
BG01761	*Amphiphysin*	26.80 ± 0.93	5.66	0.019	*AMPH*
BG01863	*Cysteine string protein*	20.50 ± 1.54	46.97	< 0.0001	*DNAJC5B*
BG02106	*CG31145*	26.40 ± 0.97	2.03	0.157	*FAM20A*
BG02109	*tartan*	26.15 ± 0.92	1.16	0.096	N/A
BG02121	*center divider*	26.80 ± 0.77	1.16	0.285	N/A
BG02676	*CG10990*	25.30 ± 1.20	16.86	< 0.0001	*PDCD4*
BG02715	*nebbish*	25.25 ± 1.27	14.41	< 0.0001	*KIF14*

The mean locomotor reactivity of the Canton S B control strain is 28.50 ± 0.20 s. Bonferroni significance threshold = 0.005. Human orthologs have homology scores of > 0.93 and Bootstrap scores of > 93%. N/A, not applicable; SE, standard error.

Mutations in each significant gene had lower levels of locomotor reactivity than the control line. Of these genes, four have been previously implicated to affect activity: *muscleblind *mutants are paralytic [36]; *Amphiphysin *[37] and *Cysteine string protein *[38] mutants are sluggish; and *nebbish *mutants are not well coordinated [39].

## Discussion

### Genetic architecture of locomotor reactivity

*D. melanogaster *exhibits a strong response to artificial selection for high and low levels of locomotor reactivity. The heritability of locomotor reactivity is fairly high for a behavioral trait (approximately 0.16). However, the genetic response to selection, as inferred from the realized heritability, was asymmetrical. Responses were much greater in the direction of decreased locomotor reactivity (heritabilities approximately 0.20) than for increased activity. Asymmetrical responses to selection are often observed for traits that are major components of fitness [29,40]. However, in this case we cannot rule out a more trivial explanation: the attenuated selection differential in the H lines. The highly reactive individuals remained active for the majority of the 45 s assay period. Indeed, we recorded the locomotor reactivity of flies from the high selection lines for assay periods of one to five minutes, and found that most flies were active throughout the assay period regardless of the duration of the assay (data not shown).

The phenotypic response to selection appears to be specific for locomotor reactivity. In particular, we did not observe correlated responses to selection for locomotor reactivity for responses to different stressors, nor for other traits involving locomotion.

Since the broad sense heritability estimated from the variation among inbred lines (*H*^2 ^= 0.52) greatly exceeds the narrow sense heritability estimated from response to selection (*h*^2 ^= 0.16), we infer that considerable non-additive genetic variance due to dominance and/or epistasis affects natural variation for this trait. We estimate the additive genetic variance (*V*_*A*_) as *V*_*A *_= *h*^2^*V*_*P *_= 3.74, where *h*^2 ^is the narrow sense heritability from divergent response to artificial selection, averaged over both replicate lines, and *V*_*P *_is the total phenotypic variance for the first 10 generations averaged over all 6 selection lines (*V*_*P *_= 23.58). If only additive genetic variance affected locomotor reactivity, we would predict the total genetic variance among the inbred lines to be 2*FV*_*A *_= 7.48, for an expected *F *= 1 after 20 generations of full sib inbreeding [29]. In contrast, the estimate of the total genetic variance among the inbred lines was *V*_*G *_= 28.14. The difference, therefore, must be due to dominance and/or epistasis.

### Transciptional response to selection for locomotor behavior

We found a large transcriptional response to selection for locomotor reactivity, with changes in expression of nearly 1,800 probe sets (approximately 9.5% of the genome) between the selection lines, using a stringent false discovery rate of 0.001. Previously, we selected replicate lines for increased and decreased copulation latency [25] and increased and decreased aggressive behavior [27]; both sets of selection lines were derived from the same initial heterogeneous base population that was used in this study. We found that the transcript abundance of over 3,700 probe sets evolved as a correlated response to selection for copulation latency [25], and over 1,500 probe sets evolved as a correlated response to selection for divergent aggressive behavior [27]. These results are in contrast to analyses of transcriptional response to selection for geotaxis behavior [23] and aggressive behavior [26], in which approximately 200 genes were inferred to exhibit differences in expression between the selection lines. The discrepancy is likely to be attributable to differences in the base population used to initiate selection. In this study, and others [25,27], the base population was derived from 60 isofemale lines recently collected from nature, while the other studies [23,26] used lines derived from severely restricted genetic bases. Classical quantitative genetics theory shows that the magnitude of direct and correlated responses to selection is directly proportional to the effective population size [41].

**Figure 6:**
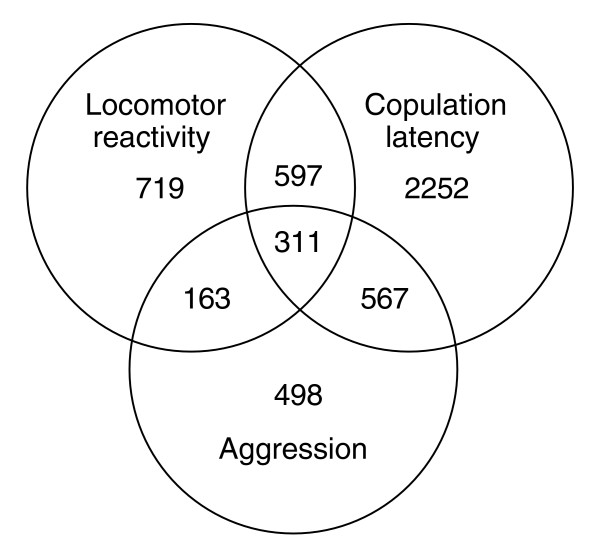
Numbers of probe sets that were significantly (*Q *< 0.001) differentially expressed between replicate selection lines selected for locomotor reactivity, copulation latency, and aggressive behavior. All selection lines were derived from the same base population.

The large number of genes exhibiting changes in transcript abundance among replicate selection lines implies that genes affecting complex behaviors are highly pleiotropic: if approximately 10% of the genome affects any one trait, the same genes must affect multiple traits. Thus, genes affecting behavior are also likely to be involved in neurogenesis, metabolism, development and general cellular processes, and many of the same genes may affect multiple behaviors. Indeed, 1,638 probe sets that were significant at a false discovery rate of *Q *< 0.001 were common between the selection lines for locomotor reactivity (this study), aggression [27], and copulation latency [25] that were all initiated from the same base population (Additional data file 6, Figure 6). We assessed whether we observed more common differentially regulated probe sets than expected by chance using χ^2 ^tests. We found 908 probe sets in common between copulation latency and locomotor reactivity (χ_1_^2 ^= 862, *P *<< 0.0001), 474 probe sets in common between aggressive behavior and locomotor reactivity (χ_1_^2 ^= 731, *P *<< 0.0001), and 878 probe sets in common between copulation latency and aggressive behavior (χ_1_^2 ^= 1,076, *P *<< 0.0001). The transcript abundance of 311 genes (Additional data file 7) was altered as a correlated response to selection for all three behaviors (χ_1_^2 ^= 2,736, *P *<< 0.0001).

**Figure 7:**
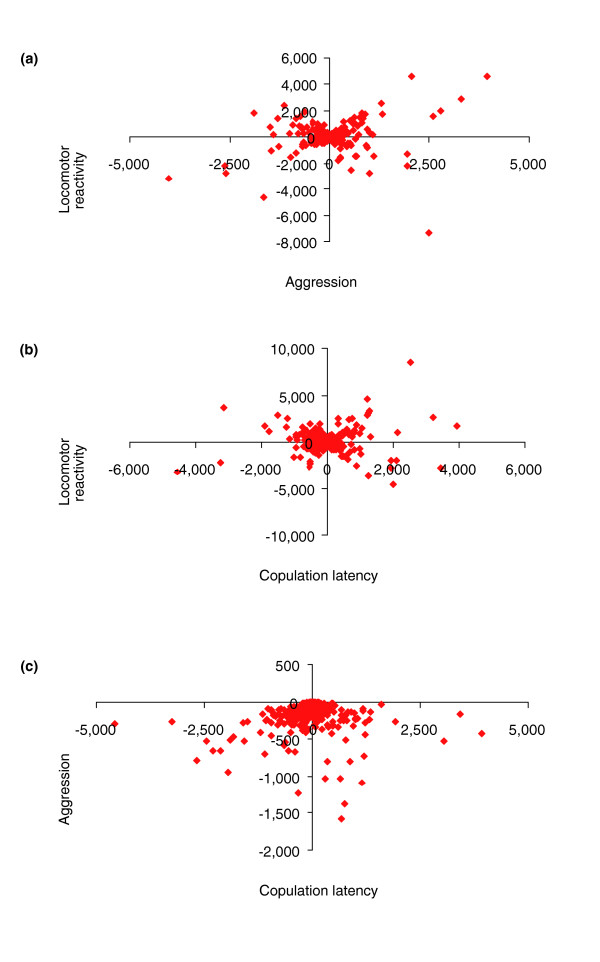
Correlations of the mean H - L difference in expression (x- and y-axes) for significant probe sets in common between two behaviors. Only probe sets that had significant contrast statements (*P *< 0.05, pooled across sexes) of H ≠ L for locomotion and aggression, and F ≠ S for copulation latency are represented. **(a) **Locomotor reactivity and aggression; **(b) **locomotor reactivity and copulation latency; **(c) **aggression and copulation latency.

The magnitude of the overlapping correlated transcriptional responses to selection for three different behaviors is astonishing. However, the patterns of the transcriptional responses to selection for the three behaviors were not correlated. We computed the correlations of the difference in mean expression between the H and L selection lines (or fast and slow lines for the case of mating behavior) for common probe sets that had significant H ≠ L contrast statements for all pairwise combinations of behaviors. The data set consisted of 655 probe sets for the comparison of copulation latency and locomotor reactivity, 580 probe sets for the comparison of mating and aggressive behavior, and 270 probe sets for the comparison of locomotion and aggressive behavior. The correlations of the mean difference in expression between H and L lines were: *r *= 0.059 ± 0.060 between mating and locomotor behavior; *r *= 0.025 ± 0.012 between mating and aggressive behavior; and *r *= 0.034 ± 0.081 between aggressive and locomotor behavior (Figure 7). Similarly, there is no association between the four categories of patterns of transcriptional response to selection for the 311 genes in common to all three behaviors (data not shown). Thus, it is not likely that the common probe sets are associated with the locomotor component of the behaviors, or with the reductions in fitness that typically occur as an unwanted side-effect of response to artificial selection, since in these cases one would expect correlated patterns of expression differences between selection lines. However, both the specific phenotypic responses to selection for the three behaviors [25,27] (this study) and the lack of correlation of patterns of transcript abundance of common co-regulated genes suggest that multiple alleles at pleiotropic genes with independent effects on different behaviors segregate in natural populations. This inference is bolstered by a recent observation that independent molecular polymorphisms at *Catecholamines up *are associated with locomotor behavior, longevity and sensory bristle number in *Drosophila *[12].

The probe sets with altered transcriptional responses to selection that were in common to at least two behaviors were enriched for genes in the *cytochrome P-450 *and *Glutathione S tranferase *gene families, genes affecting lipid metabolism, genes encoding immune/defense molecules, and genes affecting circadian rhythm and sleep (data not shown). Pleiotropic effects of genes affecting circadian rhythm, cytochrome P450s and glutathione S transferases on multiple behaviors in *Drosophila *have been implicated by other studies. Expression levels of *Pigment dispersing factor *and *cryptochrome*, both of which affect circadian rhythm, were up-regulated in lines selected for positive geotaxis, and confirmed to affect geotaxis behavior in functional tests [23]. Similarly, one member of the cytochrome P450 gene family was recently shown to affect aggressive behavior [26], and another member of this family was down-regulated in lines derived from nature with high male reproductive success [42]. The expression of many cytochrome P450 family members was altered in mated relative to virgin females [43]. Glutathione transferase genes are thought to be involved in resistance to oxidative stress [44]. Glutathione transferase genes were also over-represented in a whole genome expression study comparing natural lines with high and low levels of male reproductive success [42]. Two of these genes, *GstE5 *and *GstE1*, were among those that were differentially expressed between lines selected for locomotor behavior (this study), aggressive behavior [27] and mating behavior [25]. Functional evidence linking glutathione transferase activity to locomotor behavior comes from the observation that the locomotor defect of *Drosophila parkin *mutants is enhanced by loss-of-function mutants of *GstS1 *and reduced when *GstS1 *is over-expressed [45].

The transcriptional response to selection that we observed is attributable to genes that have causally responded to selection, and genes that are co-regulated by these genes. Since the transcriptional response to single mutations with subtle phenotypic effects can involve over 100 co-regulated genes [46], the number of selected loci causing the changes in transcript abundance between the selection lines could be rather large. It will be necessary to map the QTL causing divergence between the H and L lines in order to elucidate causal versus consequential transcriptional responses and correlated responses to selection.

### Candidate genes for locomotor behavior

Regardless of whether or not the observed changes in gene expression are causally associated with genetic divergence in locomotor behavior between the selection lines, the genes exhibiting altered expression levels are candidate genes affecting locomotor behavior. We quantified locomotor reactivity for ten mutations in candidate genes that were generated in a common co-isogenic background, and identified seven genes with mutational effects on locomotor reactivity, six of which are significant using a conservative Bonferroni correction for multiple tests. Locomotor defects have been described previously for mutations of four of these genes. *muscleblind *encodes a protein with a zinc-finger domain involved in muscle development, and mutants are paralytic [36]. The mutant allele of *muscleblind *that had reduced locomotor reactivity in this study was also associated with increased aggressive behavior [27], consistent with the above inference regarding non-directional allelic effects of pleiotropic genes on behavioral traits. *Cysteine string protein *is involved in stress response and neurotransmitter secretion, and mutants are sluggish and hypoactive [38]. *nebbish *is involved in mitotic chromosome segregation, and mutants in this gene have been characterized as not well coordinated [39]. *Amphiphysin*, which did not surpass the conservative estimate of significance, but is formally significant at the *P *< 0.05 significance level, is involved in neurotransmitter secretion and regulation of muscle contraction, and mutants have been shown to be flightless and hypoactive [37]. The remaining three genes, *CG10990*, *CG33523*, and *ken and barbie *are novel genes affecting locomotor behavior. *CG10990 *and *CG33523 *are computationally predicted genes with unknown functions, and *ken and barbie *is involved in copulation and insemination [47].

The success of these functional tests validates using expression profiling on genetically divergent lines in directed mutagenesis screens to identify genes affecting complex traits. This strategy is complementary to traditional strategies and cannot supplant them, since many key genes will not be detected as differentially expressed. Specifically, we will not detect genes that are differentially expressed at a different developmental period or if the magnitude of the difference in transcript abundance is too small to be detected, or genes affecting the trait that are not regulated at the level of transcription. Of the 82 genes with known effects on some aspect of locomotor behavior, 20 were not present on the microarray, and eight did not give a statistically significant signal (that is, were called absent). Of the 54 remaining genes, 16 were statistically differentially expressed between the selection lines at nominally significant *P *values that did not meet our conservative false discovery rate criterion, and 13 genes (*cacophony*, *Drop*, *homer*, *Shaker*, *TBP-related factor*,*turtle*, *Casein kinase IIα*, *Clock*, *cycle*, *discs overgrown*, *cAMP-dependent protein kinase1*, *shaggy *and *timeless*) were differentially expressed at *Q *< 0.001 (Additional data file 8). We identified 13 positional candidate genes affecting locomotor reactivity in our previous QTL mapping study [1]. Of these genes, only *Drop *and *turtle *showed significant differences in transcript abundance between the selected lines. These results highlight the utility of applying multiple complementary approaches to understand the genetic basis of complex traits.

Many of the genes with mutational effects on locomotor behavior are evolutionarily conserved and have human orthologs (Table 2). For example, *muscleblind *is orthologous to *Muscleblind-like Protein 1*, which encodes a triplet repeat expansion, similar to the one that causes muscular dystrophy [48]. It is thus possible that the genes and pathways affecting locomotion in *Drosophila *will elucidate corresponding mechanisms in other organisms, including humans.

## Materials and methods

### *Drosophila *stocks

Flies were reared on cornmeal/molasses/agar medium under standard culture conditions (25°C, 12:12 h light/dark cycle). Behavioral assays were conducted in a behavioral chamber (25°C, 75% humidity) between 8 am and 12 pm (2-6 h after lights on).

### Locomotor reactivity assay

Locomotor reactivity was assessed as described previously [1,12]. Briefly, we placed single 3-7 day old adult flies, collected under CO_2 _exposure, into vials containing 5 ml of standard cornmeal/agar/molasses media, and left them overnight to acclimate to their new environment. To quantify locomotor reactivity, we subjected each fly to a mechanical disturbance by tapping the vial twice against a table, and recorded the amount of time the fly is active in the 45 seconds immediately following the disturbance using a stopwatch to record movement, while a timer counts down the 45 s assay period. The measure of locomotor reactivity is a score ranging from 0 s to 45 s, denoting the total amount of activity during the assay period.

### Natural genetic variation in locomotor reactivity

Isofemale lines were established from wild-type gravid females collected at the Raleigh, NC Farmer's Market in 2003. The lines were inbred by 20 generations of full-sib inbreeding to create 340 inbred lines. Locomotor reactivity for each of the inbred lines was measured by randomly assigning the lines into blocks of approximately 25 lines; each block was tested over a 2 week period. We obtained 2 replicate measurements (*N *= 20 males and 20 females per replicate) for each inbred line. The replicates for each line were assessed on different days.

### Quantitative genetic analysis

Mixed model factorial analysis of variance (ANOVA) was used to partition variance in locomotor activity among the inbred lines, according to the model:

*Y *= μ + *Block *+ *Sex *+ *Line*(*Block*) + *Sex× Line*(*Block*) + *Rep*(*Line× Sex× Block*) + *E*

where μ is the overall mean, *B *is the random effect of block, *S *is the fixed main effect of sex, *Line*(*Block*) is the random main effect of line within each block, *Sex *× *Line*(*Block*) is the random effect of the sex by line interaction within each block, *Rep*(*Line *× *Sex *× *Block*) is the random effect of replicate, and *E *is the within-vial variance. Parentheses indicate nested effects. The total genotypic variance among lines was estimated as:

*σ*_*G*_^2 ^= *σ*_*L*(*B*)_^2 ^+ *σ*_*LS*(*B*)_^2^

where *σ*_*L*(*B*)_^2 ^is the among-line variance component and *σ*_*LS*(*B*)_^2 ^is the variance attributable to the *L *×*S *interaction. The total phenotypic variance was estimated as:

*σ*_*P*_^2 ^= *σ*_*G*_^2 ^+ *σ*_*E*_^2^

where *σ*_*E*_^2 ^is the environmental variance component. We estimated broad sense heritabilities as:

*H*^2 ^= *σ*_*G*_^2^/*σ*_*P*_^2^

We also ran reduced analyses for each sex separately. The genetic correlation (*r*_*GS*_) across sexes was calculated as:

*r*_*GS *_= *σ*^2^_*L*_/(*σ*_*LM*_*σ*_*LF*_)

where *σ*^2^_*L *_is the among-line variance component from the analysis pooled across both sexes, and *σ*_*LM *_and *σ*_*LF *_are the square roots of the among-line variance components from the analyses of each sex separately. All statistical analyses were performed using SAS procedures (SAS Institute, Cary, NC, USA).

### Artificial selection for locomotor reactivity

The base population was generated from 60 isofemale lines established from flies collected in Raleigh, NC in 1999. The isofemale lines were crossed in a round robin design (line 1 ♀ × line 2 ♂, line 2 ♀ × line 3 ♂, line 60 ♀ × line 1 ♂). Single fertilized females from each cross were placed in each of two culture bottles. The two initial bottles were split into three replicate bottles each that were arbitrarily assigned into H, L, and C groups. The six bottles were maintained by random mating for four generations to allow initial linkage disequilibrium to decay. After four generations of random mating, the following generation (G1) and all subsequent generations repeated the same procedure: 50 virgin females and males were scored from each line (H, L, and C), and the 20 most active males and females from the H lines and the 20 least active males and females from the L lines were selected as parents for the next generation. The first 20 males and females scored from the C lines were used as the C line parents. Selection was continued for 25 generations.

Estimates of realized heritability (*h*^2^) were calculated by regression of the cumulative selection response (Σ*R*) on the cumulative selection differential (Σ*S*) [29].

### Correlated responses to selection

Starvation resistance was assessed as previously described [49]. Single sex groups of ten two-day-old flies were placed in vials containing non-nutritive medium (1.5% agar and 5 ml water). Survival was scored every 8 h until all flies were dead. This assay was conducted for generations 23-25, with five replicate measurements per line per sex per generation.

Chill-coma recovery was quantified as previously described [50]. We transferred 25 3- to 7-day-old flies per line per sex per generation without anesthesia into an empty vial and placed them on ice for 3 h. The flies were then transferred to room temperature, and the recovery time was recorded as the length of time necessary for an individual to right itself and stand on its legs. The assay was performed at generations 23-25.

Ethanol sensitivity was measured using an inebriometer [51]. We aspirated 50-60 same-sex flies per line per generation into a glass column with mesh partitions, which was filled with saturated ethanol vapors. The flies lose postural control due to ethanol exposure and fall down the partitions to the bottom of the column, where they were collected at one minute intervals. The elution time was recorded as the measure of ethanol sensitivity. This assay was conducted for generations 23-25.

Copulation latency was scored as previously described [25]. For each selection line per generation, 20 pairs of 3- to 7-day-old virgin flies were aspirated into vials containing approximately 3 ml standard culture medium. The score recorded for a pair was the number of minutes from introduction to the vial until initiation of copulation. Assays were performed at generations 23-25.

Locomotor senescence was measured at generation 25. Approximately 200 2- to 4-day-old same-sex flies from each line were placed in separate bottles. The flies were transferred to fresh bottles every 2-3 days, and each week (on days 14, 21, 28, 35, 42, 49, and 56), 25 flies of each line and sex were scored for locomotor reactivity.

### Statistical analysis of correlated responses

Differences between the selection lines for the correlated traits were assessed using a nested mixed model analysis of variance (ANOVA):

*Y *= *μ *+ *Selection *+ *Line*(*Selection*) + *Sex *+ *Gen *+ *Selection*×*Sex *+ *Selection*×*Gen *+ *Line*(*Selection*)×*Sex *+ *Line*(*Selection*)×*Gen *+ *Sex*×*Gen *+ *Selection*×*Sex*×*Gen *+ *Line*(*Selection*)×*Sex*×*Gen *+ *ε*

where *Y *is the phenotypic score, *μ *is the overall mean, *Selection *is the fixed effect of the selection treatment (high activity, control, or low activity), *Line*(*Selection*) is the random effect of the replicate within each selection group, *Sex *is the fixed effect of sex, *Gen *is the fixed effect of generation, and *ε *is the error variance. A significant *Selection *term is indicative of a correlated response in the trait being tested to selection for locomotor behavior. Behavioral locomotor senescence was analyzed in the same manner, with the replacement of the *Week *term instead of *Gen *term.

### Whole genome expression profiling

At generation 25, two replicates of 12 3- to 7-day-old virgin males and females were collected from each selection line, at the same time of day that the behavioral assays were performed. Total RNA was extracted from the 24 samples (6 lines × 2 sexes × 2 replicates) using the Trizol reagent (Gibco BRL, Gaithersburg, MD, USA). Biotinylated cRNA probes were hybridized to high density *Drosophila *GeneChip 2.0 oligonucleotide microarrays (Affymetrix, Inc., Santa Clara, CA, USA)) and visualized with a streptavidin-phycoerythrin conjugate, as described in the Affymetrix GeneChip Expression Analysis Technical Manual (2000), using internal references for quantification. The quantitative estimate of expression of each probe set is the *Signal *(*Sig*) metric, as described in the Affymetrix Microarray Suite, Version 5.0.

### Microarray data analysis

The 18,800 probe sets on the Affymetrix *Drosophila *GeneChip 2.0 are represented by 14 perfect-match (PM) and 14 mismatch (MM) pairs. The *Sig *metric is computed using the weighted log(PM - MM) intensity for each probe set, and was scaled to a median intensity of 500. A detection call of present, absent, or marginal is also reported for each probe set. We excluded probe sets with more than half of the samples called absent from the analysis, leaving 11,656 probe sets. This filter retained sex-specific transcripts but eliminated probe sets with very low and/or variable expression levels [25]. On the remaining probe sets, we conducted two-way fixed effect ANOVAs of the Signal metric, using the following model:

*Y *= *μ *+ *Line *+ *Sex *+ *Line*×*Sex *+ *ε*

where *Sex *and *Line *are the fixed effects of sex and selection line and *ε *is the variance between replicate arrays. We corrected the *P-*values computed in these ANOVAs for multiple tests using a stringent false-discovery rate criterion [33] of *Q *< 0.001. We used contrast statements [25,27] to assess whether expression levels of probe sets with *L *and/or *S *× *L *terms at or below the *Q *= 0.001 threshold were significantly different between selection groups (H, C, and L) at the *P *< 0.05 level, both within each sex and pooled across sexes. GO categories were annotated using DAVID [52] (Table 1) and Affymetrix [53] and FlyBase [54] compilations (Additional data files 4 and 5).

### Functional tests of mutations in candidate genes

We tested whether mutations in ten of the candidate genes with altered transcript abundance between the selection lines affected locomotor behavior. The mutations were homozygous *P{GT1} *elements inserted within the candidate genes, and all were generated in a common co-isogenic background (Canton S, B background) [35]. Locomotor reactivity was assessed for all mutant lines using the same assay as the selection lines, but using a 30 s assay period. The ten mutant lines were tested in two blocks, with ten flies/sex/line/block. We also assessed 20 flies per sex of the co-isogenic control line (B) in each block. We used the following ANOVA model to determine whether the locomotor reactivity of the mutant lines differed significantly from the control:

*Y *= *μ *+ *Line *+ *Sex *+ *Block *+ *Line*×*Sex *+ *Line*×*Block *+ *Block*×*Sex *+ *Line*×*Sex*×*Block *+ *ε*

where *Y *is the phenotypic score, *μ *is the overall mean, *Line *is the fixed effect of the genotype (mutant or control), *Block *is the random effect of the replicate, *Sex *is the fixed effect of sex, *Line*×*Sex *(fixed), *Line*×*Block *(random), *Block*×*Sex *(random), and *Line*×*Sex*×*Block *(block) are the interaction terms, and *ε *is the variance within lines. A significant *Line *term suggests that the mutant is significantly different from the control.

## Additional data files

The following additional data are available with the online version of this paper. Additional data file [Supplementary-material S1] contains raw microarray data. Additional data file 2 gives *P *and *Q *values for all probe sets with significant (*Q *< 0.001) expression differences between selection lines. Additional data file 3 gives *P *values of significant contrasts of mean expression differences between selection lines. Additional data file 4 gives GO categories of differentially expressed genes. Additional data file 5 shows the GO categories that are over-represented by differentially expressed genes. Additional data file 6 lists probe sets common to multiple behaviors. Additional data file 7 gives *P *values of significant contrasts for probe sets that are differentially expressed between lines independently selected for locomotor, aggressive and mating behavior. Additional data file 8 shows known genes affecting locomotor behavior.

## Abbreviations

ANOVA = Analysis of Variance; C line = control selection line; GO = Gene Ontology; H line = high selection line; L line = low selection line; QTL = quantitative trait locus.

## Authors' contributions

KWJ and TFCM designed the experiment, KWJ conducted the selection experiment and assays, MAC extracted and labeled RNA for the microarray experiment, AY carried out the functional tests to putative candidate genes, TJM performed the statistical analysis of the microarray data, and KWJ and TFCM wrote the paper.

## Supplementary Material

Additional data file 1 Raw microarray data. Click here for file

Additional data file 2 *P *and *Q *values for all probe sets with significant (*Q *< 0.001) expression differences between selection lines. Click here for file

Additional data file 3 *P *values of significant contrasts of mean expression differences between selection lines. Click here for file

Additional data file 4 GO categories of differentially expressed genes. Click here for file

Additional data file 5 GO categories that are over-represented by differentially expressed genes. Click here for file

Additional data file 6 Probe sets common to multiple behaviors. Click here for file

Additional data file 7 *P *values of significant contrasts for probe sets that are differentially expressed between lines independently selected for locomotor, aggressive and mating behavior. Click here for file

Additional data file 8 Known genes affecting locomotor behavior. Click here for file
