# Supplementary material for: Quantitative genomics of locomotor behavior in Drosophila melanogaster
Source: Genome Biol. 2007 Aug 21;8(8):R172. doi: 10.1186/gb-2007-8-8-r172 (PMC2375002; doi:10.1186/gb-2007-8-8-r172)
Supplement: Additional data file 8 — Known genes affecting locomotor behavior. [file gb-2007-8-8-r172-S8.doc]

**Table S8. Genes Affecting Locomotor Behavior**

***P*<0.05; *P*<0.05;**

**Function/*Gene* *P*>0.05 *Q*>0.001 *Q*<0.001**

**Adult Locomotion**

*Aconitase* 5.93 × 10^−2^

*Actinin*  6.09 × 10^−1^

*bendless*  1.28 × 10^−1^

*beethoven* 9.47 × 10^−1^

*C141*

*C31*

*cacophony* 6.60 × 10^−4^

*calcium dependent kinase* 2.38 × 10^−2^

*Catsup* 5.87 × 10^−2^

*central complex*

*Crippled A*

*crooked legs* 2.13 × 10^−3^

*Dopa decarboxylase* 2.60 × 10^−2^

*Deadpan*

*Drop* 1.61× 10^−4^

*derailed*  6.61 × 10^−1^

*ether a gogo*

*ellipsoid body open*

*eyeless*

*flightless 1* 7.99 × 10^−1^

*giant fiber A*

*highwire* 2.57 × 10^−1^

*Hyperkinetic* 2.24 × 10^−2^

*homer*  7.68 × 10^−4^

*hitzschlag*

*Hypoactive A*

*Hypoactive C*

*inactive*

*Lim3* 8.76 × 10^−2^

*narrow abdomen*

*neither inactivation nor afterpotential E* 1.43 × 10^−1^

*no bridge*

*no receptor potential A*  1.92 × 10^−1^

*ocelliless* 3.88 × 10^−1^

*overheated*

*P-25*

*pale*

*reduced eye*

*Sema 2A* 4.32 × 10^−2^

*Shaker* 8.01× 10^−4^

*shuttlecraft* 7.14 × 10^−2^

*single minded* 1.61 × 10^−1^

*Sphingosine kinase 2* 2.36 × 10^−2^

*slow receptor potential*

*stoned A*

*tan*

*TBP related factor* 3.56 × 10^−4^

*thickened aristae*

***P*<0.05; *P*<0.05;**

**Function/*Gene* *P*>0.05 *Q*>0.001 *Q*<0.001**

**Adult Locomotion**

*turtle* 2.36 × 10^−5^

*uncoordinated like*

**Locomotor Rhythm**

*Beadex* 5.32 × 10^−2^

*Casein kinase subunit II α*  1.09 × 10^−3^

*Clock* 6.48 × 10^−10^

*CrebB*

*cycle*  4.10 × 10^−4^

*discs overgrown* 1.16 × 10^−3^

*disconnected* 5.46 × 10^−1^

*dunce* 7.75 × 10^−1^

*ebony* 1.16 × 10^−1^

*Fmr1* 5.09 × 10^−3^

*lark* 2.75 × 10^−2^

*Neurofibromin 1* 2.50 × 10^−2^

*Open rectifier K+ channel 1* 5.13 × 10^−1^

*Pigment dispersing factor* 8.46 × 10^−1^

*period* 3.82 × 10^−2^

*cAMP dependent protein kinase-1*  3.39 × 10^−9^

*cAMP dependent protein kinase-R2* 1.24 × 10^−1^

*shaggy* 3.32 × 10^−5^

*supernumerary limbs*  3.76 × 10^−2^

*timeless* 1.49 × 10^−3^

*vrille* 1.01 × 10^−2^

**Larval Locomotion**

*Adh transcription factor 1* 2.25 × 10^−1^

*Chaser*

*draper* 2.49 × 10^−3^

*Ecdysone Receptor* 1.55 × 10^−1^

*foraging* 4.73 × 10^−2^

*Glutamic acid decarboxylase 1* 3.61 × 10^−3^

*neuropeptide F* 5.07 × 10^−2^

*pickpocket*

*scribbler* 5.87 × 10^−3^

*slowmo* 6.45 × 10^−2^

*synaptotagmin*

Genes in red font were not present on the array, and genes in blue font were called Absent. Entries in the table are *P*-values from tests of significance of expression differences between the selection lines.
